# Interplay of Metallome and Metabolome in Amyotrophic Lateral Sclerosis: A Study on Cerebrospinal Fluid of Patients Carrying Disease-Related Gene Mutations

**DOI:** 10.1021/acschemneuro.3c00128

**Published:** 2023-08-23

**Authors:** Nikolay Solovyev, Marianna Lucio, Jessica Mandrioli, Sara Forcisi, Basem Kanawati, Jenny Uhl, Marco Vinceti, Philippe Schmitt-Kopplin, Bernhard Michalke

**Affiliations:** † Analytical BioGeoChemistry Research Unit, Helmholtz Center Munich—German Research Center for Environmental Health GmbH, Ingolstädter Landstr. 1, 85764 Neuherberg, Germany; ‡ Department of Biomedical, Metabolic and Neural Sciences, University of Modena and Reggio Emilia, 41125 Modena, Italy; § Department of Neurosciences, Azienda Ospedaliero Universitaria di Modena, 41126 Modena, Italy; ∥ CREAGEN Research Center of Environmental, Genetic and Nutritional Epidemiology, Department of Biomedical, Metabolic and Neural Sciences, University of Modena and Reggio Emilia, 41125 Modena, Italy

**Keywords:** amyotrophic lateral sclerosis, disease-related mutations, metabolomics, metallomics, brain steroids

## Abstract

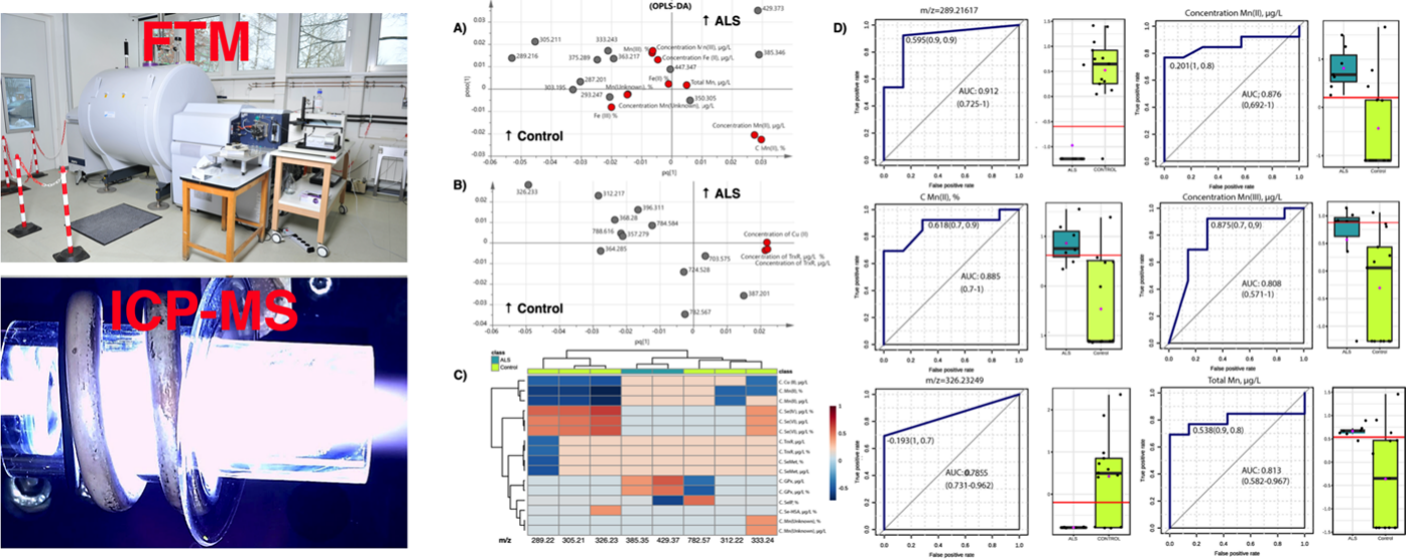

Amyotrophic lateral sclerosis (ALS) is a lethal progressive neurodegenerative disease, characterized by a loss of function of upper and lower motor neurons. This study aimed to explore probable pathological alterations occurring in individuals with ALS compared to neurologically healthy controls through the analysis of cerebrospinal fluid (CSF), a medium, which directly interacts with brain parenchyma. A total of 7 ALS patients with disease-associated mutations (*ATXN2*, *C9ORF72*, *FUS*, *SOD1*, and *TARDBP*) and 13 controls were included in the study. Multiple analytical approaches were employed, including metabolomic and metallomics profiling, as well as genetic screening, using CSF samples obtained from the brain compartment. Data analysis involved the application of multivariate statistical methods. Advanced hyphenated selenium and redox metal (iron, copper, and manganese) speciation techniques and nontargeted Fourier transform ion cyclotron resonance mass spectrometry-based metabolomics were used for data acquisition. Nontargeted metabolomics showed reduced steroids, including sex hormones; additionally, copper and manganese species were found to be the most relevant features for ALS patients. This indicates a potential alteration of sex hormone pathways in the ALS-affected brain, as reflected in the CSF.

## Introduction

Amyotrophic lateral sclerosis (ALS) is a neurodegenerative disease of motor neurons; the loss of function of upper and lower motor neurons causes progressive muscular atrophy and death usually within a few years after the diagnosis.^1^ The incidence worldwide is estimated at 2–3 per 100,000 people, with a slightly greater incidence and prevalence in males.^2,3^ Although major advances in ALS genetics provided fundamental insights into motor neuron degeneration,^4^ genetic ALS remains poorly understood, with etiology not fully clear. Depending on studies’ accuracy, familial ALS covers ca. 5% of cases, with the majority of patients considered as sporadic because they occur randomly throughout the population.^5^ Several factors have been called into question in the genesis of ALS and the “multistep” pathogenetic model, which suggests a view of the disease as a crossroad among genetic, neurometabolic, and environmental factors, represents a fascinating model of interaction, mostly accepted for many neurodegenerative diseases.^6,7^

If genetic advances in the disease had revolutionized ALS understanding, environmental factors have been widely studied but frequently led to conflicting results: which risk factors may contribute to disease onset and progression have not been identified so far. Among the most studied environmental factors, exposure to heavy metals including lead, mercury, and selenium,^8,9^ a history of physical trauma/injury, electric shock, and previous exposure to organic solvents^10^ and pesticides^11,12^ were found to be variably associated with the disease, supporting multifactorial and multipathway etiology of ALS.

Metabolomics, that is, the study of the wide set of metabolites, in the biological media of ALS patients is a valuable tool for the study of disease pathology and a search for new potential biomarkers and therapeutic targets. The characterization of the metabolome allows for uncovering disease-specific signatures, which facilitates subgroup stratification and provides new insights into dysregulated biochemical pathways in the affected tissue.^13^ Up to now, several such studies have been undertaken with respect to ALS.^14,15^ For instance, Kumar et al.^16^ used proton nuclear magnetic resonance (^1^H NMR) to examine 30 serum samples from ALS patients, finding elevated quantities of glutamate, β-hydroxybutyrate, acetate, acetone, and formate and decreased glutamine, histidine, and *N*-acetyl derivatives compared to healthy controls (*n* = 25). Gray et al.^13^ used ^1^H NMR for profiling metabolites in cerebrospinal fluid (CSF) samples of ALS patients (*n* = 41): glucose, lactate, citric acid, and ethanol were found as discriminating metabolites that are elevated in ALS compared to controls (*n* = 14). Wuolikainen et al.^17^ applied gas chromatography coupled to time-of-flight mass spectrometry (GC-TOF-MS) for the metabolite profiling of CSF; the authors reported that sporadic ALS patients had a heterogeneous metabolic signature in the CSF, while familial ALS without superoxide dismutase-1 gene (SOD1) mutation was found to form a separate homogeneous group—glutamate and glutamine were reduced. Ultrahigh-performance liquid chromatography–MS was used to describe CSF metabolites in ALS individuals by Blasco et al.^18^

Different groups of metabolites seem to be implemented in ALS pathology, including lipophilic compounds of different groups, e.g., membrane lipids and metabolites of the arachidonic acid pathway,^19,20^ steroid and steroid-like compounds,^21,22^ and so forth. Emerging research has highlighted the significant role that metals and their disrupted homeostasis play in various neurodegenerative conditions. For instance, aluminum or non-ceruloplasmin bound copper—the so-called free or exchangeable copper—was associated with the pathogenesis of Alzheimer’s disease or manganese with the development of Parkinson’s disease symptoms.^23,24^ Extensive investigation on the potential association of metal exposure and metal dyshomeostasis with neurodegenerative disorders primarily centers around evaluating metal levels in different biological samples (blood, serum, plasma, nail, and hair). Nevertheless, the findings of these studies often yield conflicting results.^25^ It has been shown that the analysis of metals and their species in CSF provided a more stringent insight into the associations between metal species changes and neurodegenerative conditions. Importantly, epidemiological studies mostly disregard the effect of metal species (“metallomics metabolites”) on the “classical” small organic molecule metabolites and vice versa. This is probably due to the lack of instrumental capabilities to simultaneously conduct metabolomics and metallomics studies on the same set of samples. Individual laboratories often specialize in either metabolomics or metallomics, leading to a potential limitation in comprehensive investigations that encompass both aspects. Metal species may affect different metabolic pathways involved in neurodegeneration, including, first, lipid peroxidation and inflammation, both influencing directly and through affecting protein aggregation and enzymatic activity.^26−29^

The current study aimed to investigate the small molecular weight metabolites in the CSF of ALS individuals with disease-associated gene mutations in comparison to matched controls and evaluate the potential interplay between CSF metabolome and metallome for a better understanding of the underlying pathological mechanisms. Particularly, selenium (Se) species appear to be either selenoproteins like glutathione peroxidases (GPX), selenoprotein P (SELENOP), or thioredoxin reductases (TRXND), thus are direct actors in promoting neurodevelopment and protection against oxidative stress, or they are Se-amino acids or inorganic Se species being shown to be detrimental in the frame of ALS. Metallic redox species also directly affect or even disrupt regular cell functions and can cause cellular dysfunction followed by lipid peroxidation, being in turn reflected in classical metabolomics studies.

The case–control study was undertaken using the same set of CSF samples, which has been analyzed to examine the potential role of Se and redox-active metals (copper—Cu, iron—Fe, and manganese—Mn) in ALS.^4,30^ We hypothesized an interaction between genetic and environmental/metabolic factors in this instance, a topic so far not investigated in such patients but that finds support from the current overall evidence about ALS etiology.^31^

## Results

Table 1 presents the key characteristics of the participants with ALS, including age and sex data. Additional information regarding the age and sex data of the control group can be found in Table S1 (Supporting Information). All samples were collected solely for diagnostic purposes (see also the Materials and Methods section), ensuring that the ALS patients included in the study did not exhibit any complications associated with the disease or therapy. The small available genetic ALS cohort contains five different disease-related mutations, where four mutations occur once each, while the *C9ORF72* mutation occurs three times. Due to the small sample number per mutation, the ALS cases were compared to the controls as a whole group.

**Table 1:** Characteristics of the ALS Patients with Disease-Related Mutations, Included in the Current Study (F—Female; M—Male)^30^^a^

gene mutation	disease type	sex	age at ALS onset, years	type of onset	disease phenotype	cognitive impairment	survival, months
*C9ORF72*	sporadic	M	52	bulbar	pyramidal	no	24
*C9ORF72*	familial	M	45	spinal	classic	no	42
*C9ORF72*	sporadic	F	56	spinal	classic	no	66
*ATXN2*	familial	F	64	spinal	classic	no	9
*SOD1* (p.N66T)	sporadic	M	55	spinal	flail leg	no	82
*FUS* (P525L)	sporadic	F	12	spinal	classic	no	14
*TARDBP* (A382T)	sporadic	F	50	spinal	classic	no	24

a Age- and sex-control matching of the cases is presented in Table S1 (Supporting Information).

The pure concentration data of selenium, copper, iron, and manganese species in the CSF of ALS patients and controls have been reported already previously,^4,30^ but here, we correlate them with the newly presented metabolites data from electrospray ionization Fourier transform ion cyclotron resonance MS (FT-ICR-MS). To determine the association of each (metallo)—metabolite with the two sample groups, orthogonal partial least square model discriminant analysis (OPLS-DA) was employed. Tables 2 and 3, respectively, summarize all metabolites and metal chemical species identified in CSF samples of both groups, controls and ALS. Based on OPLS-DA analysis, Tables 2 and 3 report the loading values and derived compound association to either the control or ALS group, providing insights into the discriminatory power of each variable.

**Table 2:** Most Important Compounds Related to the Control-ALS Groups Identified Using OPLS-DA Analysis^a^

formula of neutral compound	putative KEGG/HMDB identity	alteration in ALS disease	loadings values comp. 1	loadings values comp. 2	theoretical ion Mass, Da	experimental ion Mass, Da	ion type	ion mass error (ppm)	metabolite category
C_14_H_18_O_4_	ubiquinone Q1	↓	–0.02	0.022	251.127786	251.127809	[M + H]^+^	–0.09	lipids (lipophilic compounds)
C_18_H_28_O_2_	(6*Z*,9*Z*,12*Z*,15*Z*)-octadecatetraenoic acid	↓	–0.046	0.034	277.216206	277.216213	[M + H]^+^	–0.03	lipids
C_19_H_24_O_2_	boldione	↓	–0.021	0	285.184906	285.184933	[M + H]^+^	–0.10	steroids (exogenic)
C_19_H_26_O_2_	androst-4-ene-3,17-dione (androstenedione)	↓	–0.013	–0.001	287.200556	287.200538	[M + H]^+^	0.06	steroids
C_19_H_28_O_2_	testosterone	↓	–0.027	0.009	289.216206	289.216167	[M + H]^+^	0.14	steroids
C_19_H_32_O_2_	3β,17β-dihydroxyetiocholane	↓	–0.016	–0.007	293.247506	293.247490	[M + H]^+^	0.05	steroids
C_19_H_26_O_3_	2-methoxy-17β-estradiol	↓	–0.03	0.007	303.195471	303.195502	[M + H]^+^	–0.10	steroids
C_19_H_28_O_3_	3α,16β-dihydroxyandrostenone	↓	–0.034	0.022	305.211121	305.211116	[M + H]^+^	0.02	steroids
C_17_H_29_NO_4_	2-trans,4-cis-decadienoylcarnitine	↓	–0.001	0.036	312.216935	312.216945	[M + H]^+^	–0.03	lipids (carnitine)
C_21_H_30_O_2_	progesterone	↓	–0.036	0.029	315.231856	315.231922	[M + H]^+^	–0.21	steroids
C_18_H_31_NO_4_	10-nitrolinoleic acid	↓	–0.019	0.017	326.232585	326.232489	[M + H]^+^	0.29	lipids (nitrosylated fatty acids)
C_21_H_32_O_3_	16α-hydroxypregnenolone	↓	–0.02	0.011	333.242421	333.242539	[M + H]^+^	–0.35	steroids
C_22_H_39_NO_2_	*N*-(8*Z*,11*Z*,14*Z*-icosatrienoyl)-ethanolamide	↓	–0.025	0.023	350.305355	350.305320	[M + H]^+^	0.10	steroids
C_24_H_36_O_2_	(4*Z*,7*Z*,10*Z*,13*Z*,16*Z*,19*Z*)-docosahexaenoic acidethylester	↓	–0.006	–0.002	357.278806	357.278721	[M + H]^+^	0.24	lipids (fatty acids)
C_21_H_30_O_5_	poststerone	↓	–0.026	0.022	363.216601	363.216667	[M + H]^+^	–0.18	steroids
C_22_H_37_NO_3_	leukotriene B4 dimethylamide	↓	–0.027	0.019	364.28462	364.284601	[M + H]^+^	0.05	lipids (arachidonic acid)
C_21_H_37_NO_4_	3,5-tetradecadiencarnitine	↓	–0.011	0.007	368.279535	368.279569	[M + H]^+^	–0.09	lipids (carnitine)
C_24_H_38_O_3_	3-oxo-5β-cholanic acid	↓	–0.017	0.019	375.289371	375.289364	[M + H]^+^	0.02	steroids
C_23_H_39_NO_3_	arachidonoyl serinol	↓	–0.017	0.019	378.30027	378.300221	[M + H]^+^	0.13	lipids (arachidonic acid)
C_21_H_37_NO_5_	3-hydroxy-5,8-tetradecadiencarnitine	↓	–0.002	0.035	384.27445	384.274396	[M + H]^+^	0.14	lipids (carnitine)
C_27_H_44_O	δ7,24-cholestadien-3β-ol	↓	–0.03	0.017	385.346491	385.346533	[M + H]^+^	–0.11	steroids
C_19_H_30_O_8_	corchoionoside C	↓	–0.012	0.022	387.201346	387.201341	[M + H]^+^	0.01	lipids (fatty acyl glycoside)
C_23_H_41_NO_4_	9,12-hexadecadienoylcarnitine	↓	–0.033	0.013	396.310835	396.310874	[M + H]^+^	–0.10	lipids (carnitine)
C_29_H_48_O_2_	3β-hydroxy-4β-methyl-5α-cholest-7-ene-4α-carbaldehyde	↓	–0.024	0.015	429.372706	429.372719	[M + H]^+^	–0.03	steroids
C_28_H_46_O_4_	3-dehydroteasterone	↓	0.014	–0.002	447.346886	447.346964	[M + H]^+^	–0.17	steroids
C_39_H_79_N_2_O_6_P	SM(d18:0/16:1(9*Z*))	↑	0.012	–0.018	703.574851	703.574770	[M + H]^+^	0.11	lipids (cell membrane)
C_41_H_74_NO_7_P	PE(18:3(6*Z*,9*Z*,12*Z*)/P-18:1(11*Z*))	↑	0	–0.022	724.527567	724.527817	[M + H]^+^	–0.34	lipids (phospholipid)
C_42_H_82_NO_8_P	PC(14:0/20:1(11*Z*))	↑	0.024	0.022	782.567027	782.566823	[M + Na]^+^	0.26	lipids (cell membrane)
C_44_H_82_NO_8_P	PC(14:1(9*Z*)/22:2(13*Z*,16*Z*))	↑	0.017	0.008	784.585082	784.584752	[M + H]^+^	0.42	lipids (phospholipid)
C_44_H_86_NO_8_P	PC(14:0/22:1(13*Z*))	↑	0.006	–0.003	788.616382	788.616488	[M + H]^+^	–0.13	lipids (phospholipid)

a When the alteration decreases in ALS, it means that it is increasing in the control group and vice versa.

**Table 3:** Metallomics Species Correlated Positively in ALS with the Attribution of the Control Group^a^

metallomics species	alteration in ALS disease	loading values comp. 1	loading values comp. 2
concentration of Se(IV), %	↓	–0.0226	0.0198
concentration of Se-HSA, μg/L	↓	–0.0134	0.0162
concentration of Se-HSA, %	↓	–0.0142	0.0162
concentration of Se(VI), %	↓	–0.0126	0.0113
concentration of Se(VI), μg/L	↓	–0.0108	0.0221
concentration of Se(VI), μg/L	↓	–0.0120	0.0119
Fe(III), %	↓	–0.0174	–0.0042
concentration Mn(unknown), μg/L	↓	–0.0097	0.0091
Mn(unknown), %	↓	–0.0092	0.0105
concentration of SELENOP, μg/L	↓	–0.0004	–0.0096
concentration of SELENOP, %	↓	–0.0056	–0.0231
concentration Fe(II), μg/L	↓	–0.0051	0.0019
Mn(III), %	↓	–0.0063	0.0096
concentration Mn(III), μg/L	↓	–0.0064	0.0094
Fe(II), %	↓	–0.0021	–0.0030
concentration Mn(II), %	↑	0.0232	–0.0241
concentration Mn(II), μg/L	↑	0.0213	–0.0232
concentration of Cu(II), μg/L	↑	0.0166	–0.0090
concentration of TRXND, %	↑	0.0211	0.0106
concentration of TRXND, μg/L	↑	0.0207	0.0103
concentration of GPX, μg/L	↑	0.0076	0.0182
concentration of GPX, %	↑	0.0053	0.0146
total Se, μg/L	↑	0.0055	0.0110
concentration of SeMet, %	↑	0.0064	0.0088
concentration of SeMet, μg/L	↑	0.0064	0.0087
total Mn, μg/L	↑	0.0033	–0.0039

a Se(IV)—selenite; Se-HSA—selenized human serum albumin; Se(VI)—selenate; Fe(III)—trivalent iron; Mn (unknown)—unidentified manganese species; SELENOP—selenoprotein P; Fe(II)—divalent iron; Mn(III)—trivalent manganese; Mn(II)—divalent manganese; Cu(II)—divalent copper; TRXND—thioredoxin reductase; GPX—glutathione peroxidase; total Se—total selenium concentration; SeMet—selenomethionine; total Mn—total manganese concentration.

**Figure 1:**
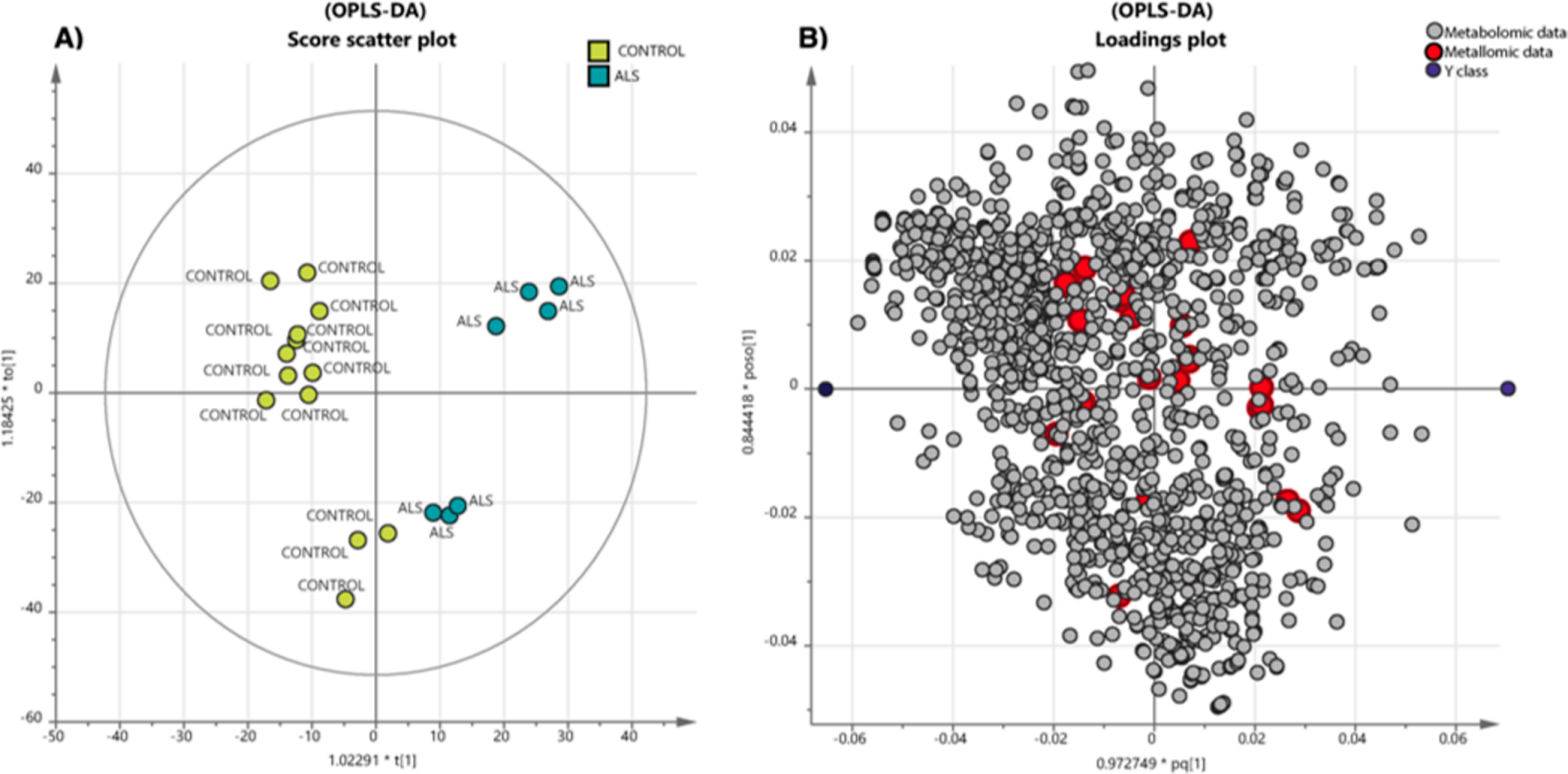
(A) Score scatter plot: The model shows separation between the ALS samples (case) vs control (*R*^2^(*Y*) = 0.84, *Q*^2^(cum) = 0.46 for the first two valid components, *p* = 0.043 for the cross-validation ANOVA). (B) Loading plot that identifies which variables have the largest contribution on the two first components (group discrimination). The red dots represent the metallomics variables, the gray ones are the metabolites. The blue dots stand for the different classes: on the left, there is the control class and, on the right, there are the cases.

Figure 1 presents a score scatter plot, which serves as the primary output of the OPLS-DA analysis conducted on the entire dataset. The plot clearly reveals that the first component (represented on the *x*-axis) effectively distinguishes the samples into two distinct groups: controls and ALS patients. The change in the identified metabolites for the ALS individuals is shown in Table 2. The variables with the highest loading values play a crucial role in characterizing the distinct clusters formed by the groups. Their role could contribute to the explication of the main differences between case and control. These variables, when associated with specific biological compounds, provide valuable insights into the primary differences between the cases and controls. Consequently, all the molecules were submitted to specific databases, namely, KEGG and/or HMDB databases. Generally, we observed a reduced diversity of metabolites in the CSF of ALS individuals compared to controls (Supporting Information, Table S2).

**Figure 2:**
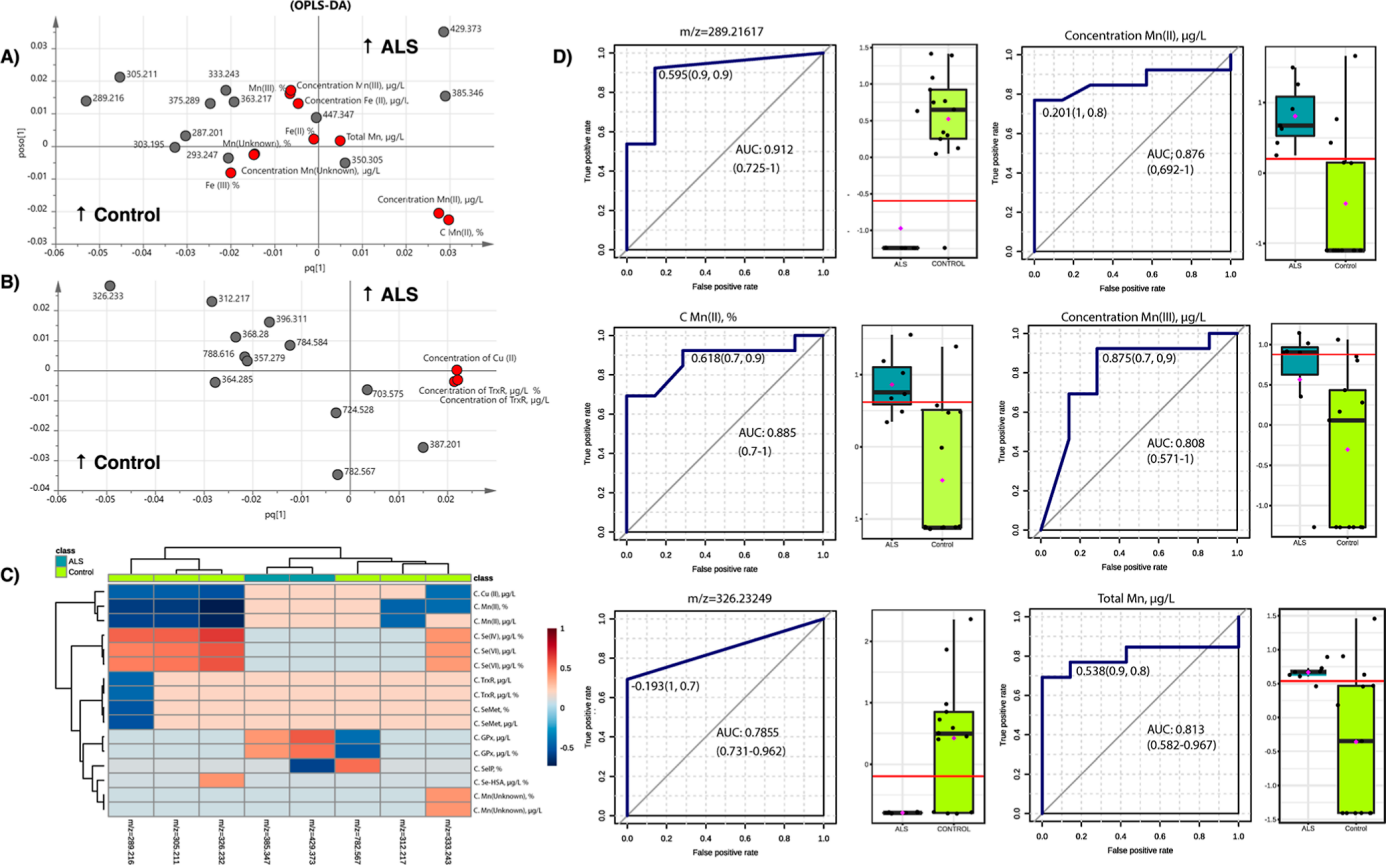
(A) DB assignment to *hormones* related to Fe and Mn species and (B) DB assignment to *lipids* related to copper and iron. The plots are a zoom-out of the loadings plot, in which we could detect the main mutual relations between the different data (metallomics and metabolomics). (C) Heatmap based on the correlation coefficients that relate the main metallomics variables with some *m*/*z* value divided for the class (control vs ALS). (D) Selection of variables most discriminative between the two groups, AUC > 0.8. The identification of the *m*/*z* according to Table 2.

In Figure 2, a closer examination of the loadings is provided, focusing on specific aspects, (A) and (B). Particularly, we highlight all mass-to-charge (*m*/*z*) values assigned to hormones that exhibit a stronger association with Fe and Mn (Figure 2A). In Figure 2B, all the loadings that express the relations between lipids and copper and iron are presented. In Figure 2C, a heat map is employed to visually represent both the positive and negative correlation relationships. In the top left of the picture, the region related to the controls, we could reveal a strong association between concentrations of Fe(II), Mn(II), and Mn(II)% Mn with male sex hormones testosterone and 3α,16β-dihydroxyandrostenone (*m*/*z* 289.216 and 305.211, respectively) and lipid oxidation product 10-nitrolinoleic acid (*m*/*z* 326.232), while they correlated with the concentration of Se(IV) and relative (%) and absolute concentrations of Se(VI). For the ALS group, we observed a moderate correlation between sterol lipid 3β-hydroxy-4β-methyl-5α-cholest-7-ene-4α-carbaldehyde (*m*/*z* 429.373) and relative contents of SELENOP and GPX concentrations, respectively (Figure 2C).

**Figure 3:**
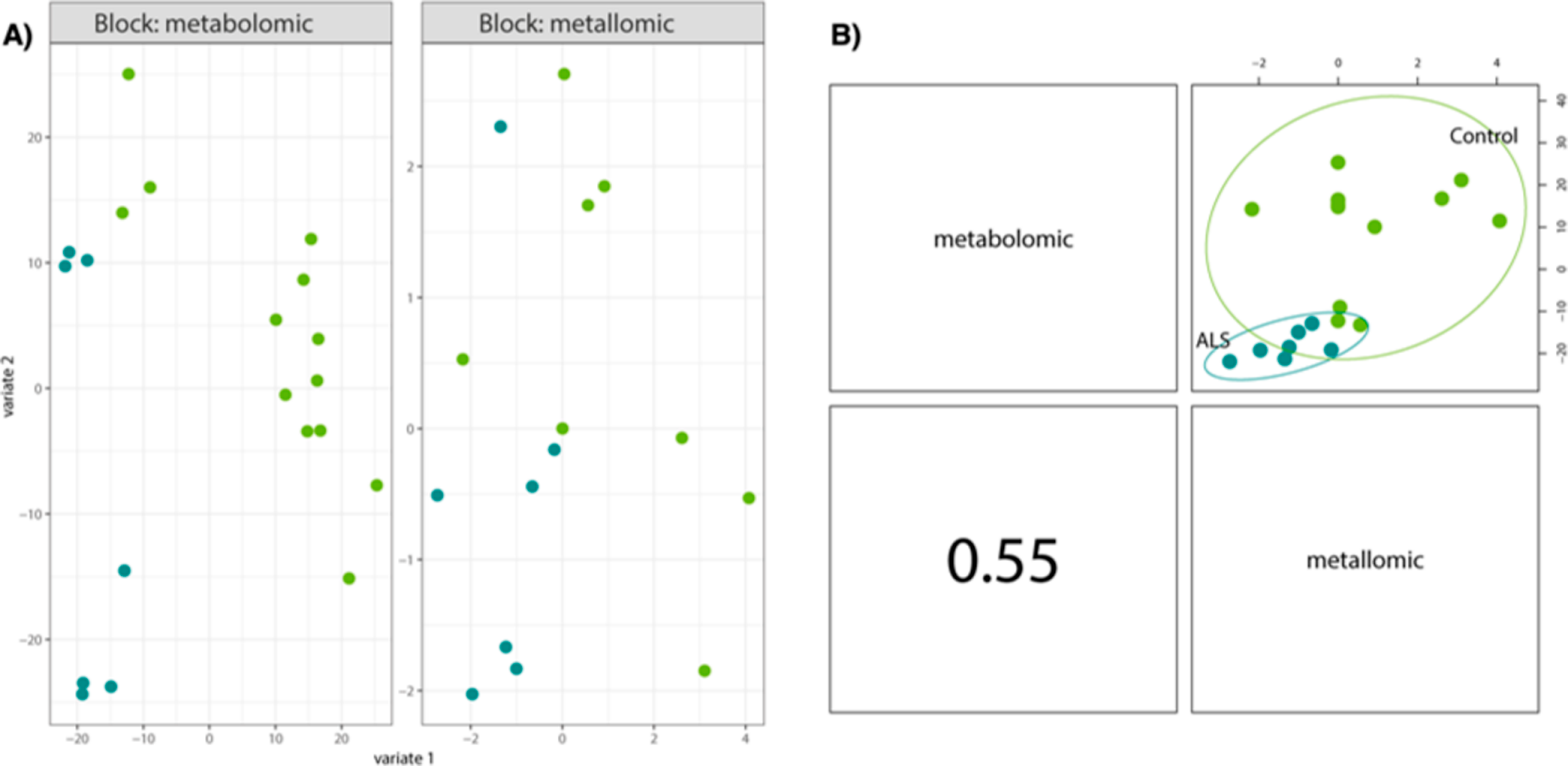
Block splsda analysis. It reveals the relation between the two different datasets (A) and the separation of the two groups in the different datasets (B).

Figure 2D presents the plot of the most discriminant variables, with an area under the curve (AUC) greater than 0.8, which effectively differentiates between the control and ALS groups. These variables exhibit strong discriminatory power, highlighting their potential as key markers for distinguishing the two groups. Figure 3 provides an explanation for the relationship between the two datasets. It illustrates that both datasets, defined as distinct blocks, possess the ability to effectively separate the two different groups (control and ALS). Both datasets, defined in blocks could separate the two different groups.

## Discussion

The integration of metallomics and metabolomics has been proven to offer a profound understanding of the interrelationships between elements and metabolites at a molecular level. This approach has previously been demonstrated in the analysis of CSF samples from patients with Parkinson’s disease.^32,33^ The method was demonstrated to be highly promising in combining different types of data sets. In this paper, these data were derived from classical FT-ICR-MS-based metabolomics and inductively coupled plasma MS (ICP–MS)-based chemical speciation approaches, particularly from seleno-metabolite data since Se is a neuroprotective element of which, however, some compounds were associated with ALS^34^ and the data of redox-metal species, the latter associated with oxidative stress.^35^

Importantly, the whole set of metabolites in the CSF is way too diverse to be fully detected using a single technique, thus, comprehensive metabolomics studies are based on the combination of several techniques since some groups of metabolites are more suited for some techniques; the use of all available techniques in the same study is not possible due to financial, technical, and analytical constraints.^18^ This complicates the comparison of different studies and the translation of the metabolomics approach to clinical applications, making the use of an adequate statistical evaluation indispensable.

In this study, we examined CSF samples from patients carrying *SOD1*, *FUS*, and *TARDBP* gene mutations and *C9ORF72* and *ATXN2* expansion, where we already found an increased SeMet content in the CSF^30^ as well as the potential role of copper.^4^ Altogether, these findings showed a biological agreement on a possible mechanism of toxicity related to ALS, namely, an impairment of protective mechanisms against oxidative stress, prooxidant, mitochondrial damage, and lipid peroxidation.

To this last point, the use of a rarely available matrix, such as CSF, in this study represents a strong suit. Metals and metabolites can be found in altered concentrations in various human biofluids (blood, serum, urine, etc.), and among these, CSF, due to its proximity to the central nervous system and relative poverty of other elements, is particularly suitable for studying neurodegenerative diseases.^36^ It is also likely that the metabolic changes, induced by toxin exposure within the brain, are reflected in the CSF.^37,38^ With this further study, we aimed at evaluating the interactions between metals and metabolites in ALS patients and controls in order to identify new pathogenetic elements and possible therapeutic targets.

The presence of a lower number of metabolites in ALS CSF is in line with previous reports,^39^ indicating potentially reduced brain metabolism in ALS reflected in CSF. The assigned metabolites showed certain alterations in the CSF of ALS patients, especially, steroids (Table 2) as well as some lipophilic molecules such as membrane lipids or arachidonic acid pathway metabolites.

The endogenic sterols were associated with their presence in the CSF of the control group, which may indicate the deprivation of their pathways in the ALS brain. These are as follows: androstenedione, 3β,17β-dihydroxyetiocholane, 2-methoxy-17β-estradiol, 3α,16β-dihydroxyandrostenone, progesterone, poststerone, 3-oxo-5β-cholanic acid, 3β-hydroxy-4β-methyl-5α-cholest-7-ene-4α-carbaldehyde, and δ7,24-cholestadien-3β-ol, which involve, first of all, androgens and estrogens, as well as steroids of other groups. Androstenedione is a degradation product form of the primary brain neurosteroid dehydroepiandrosterone, which was shown to be formed in, e.g., Alzheimer’s disease brain (post mortem) through oxidative-stress-mediated mechanisms,^40^ including mitochondrial processes.^41^ The production of neurosteroids was also noted through iron-associated mechanisms.^42−44^ Iron homeostasis is known to be impaired in ALS,^45^ so other redox-active metals (Cu and Mn) as well as selenium and redox-active selenoproteins (e.g., GPX type 4^46^) may be involved. Cu, being the key element in activated ceruloplasmin, is known to be essential for oxidizing the pro-oxidative Fe^2+^ to redox-inactive Fe^3+^, thus reducing potential ROS generation. Manganese found to be associated with ALS samples (Table 3), in turn, has been proven to promote oxidative stress and is implicated in neurodegenerative conditions.^47^ Besides, we observed some association of Mn species with steroids (Figure 2A) as well as correlations of Mn species with sex hormones and other steroids (Figure 2C). Nevertheless, their exact impacts on ALS remain elusive.

As far as steroids are concerned, they are known to be implicated in ALS as neuroprotective and neurotrophic agents acting through their nuclear receptors or independently from them. Steroids include a wide variety of compounds including sex hormones that have been claimed as responsible for sex differences in ALS.^48^ A study of sex hormones in males and females may be an intriguing perspective of further insight into the role of steroids in ALS pathology. In this study, the number of specimens was too small to test for any sex differences.

Among steroids, a neuroprotective role has been attributed to estrogens, aminosteroids, testosterone, and progesterone,^49,50^ the compounds that were found to be affected in the CSF of ALS individuals in the current study. Estrogens can exert neuroprotective activity based on antioxidant activity, interaction with mitogen-activated protein kinase, cyclic AMP pathways, and with the activity of NF-κB, and mediate cytoskeleton dynamics through interacting with Rho-GTPases and NADPH oxidase complex.^51^ Glucocorticoid compounds, like 21-aminosteroids, have high antioxidant power and prevent lipid peroxidation. They increase GFAP expression, suppress nitric oxide by decreasing NADPH-diaphorase, and attenuate the aberrant expression of both GAP-43 protein and mRNA in Wobbler motoneurons that are related to denervation and muscle atrophy.^52^

Although there are a few human studies on ALS and steroids, several shreds of evidence come from different animal models of the disease. In Drosophila models, decreased expression of TDP-43 determined the cytoplasmic accumulations of the ecdysteroid receptor (EcR) and a failure to switch EcR-dependent gene programs from a pupal to adult pattern sustaining the hypothesis that TDP-43 loss of function causes neuronal loss due to defective steroid receptor-mediated gene program switching in *Drosophila melanogaster*.^53^

Functional studies in the SOD1G93A transgenic mice showed that estradiol delays disease onset and progression and increases survival.^54^ Spinal motor neurons displayed a high density of nuclear androgen receptor (AR), estrogen receptor (ER)α, ERβ, and progesterone receptor that was differentially expressed by sex,^55^ and nandrolone treatment markedly enhanced motoneuron loss and astrocytic activation.^56^ Also, after orchiectomy,^57^ whereas progesterone treatment delayed the disease progression, reduced mutant SOD1 protein levels and motoneuronal death through activation of autophagy were observed.^49^

Another animal model (Wobbler mice) showed a hypothalamic–pituitary–gonadal hypoactivity, and testosterone treatment induced a reduction of AR, ERα, and aromatase and an increase of Sigma-1 receptor mRNAs, with a reduction in neuroinflammation and oxidative/nitrosative stress together with a slowing down the disease progression.^50^ Also, treatment with allopregnanolone alleviated the alteration of several markers of neurodegeneration (nitric oxide synthase activity, motoneuron vacuolation, MnSOD immunoreactivity, brain-derived neurotrophic factor, and TrkB mRNAs, p75 neurotrophin receptor and cell survival or death signals) and improved muscle performance.^58^ On the contrary, the same administration of nontargeted glucocorticoids in mice did not alter disease progression, maybe due to poor CNS delivery. In fact, CNS-targeted, liposomal-encapsulated glucocorticoid inhibited CNS inflammatory response, reduced motor neuron loss, and T2 hyperintensity on MRI of SOD1G93A mice.^59^ This further highlights the importance of studying fluids in proximity to the CNS, given the high impermeability of the blood–brain barrier that makes serum and plasma determination not always representative of CNS pathomechanisms.

The results on sex hormones and other steroids (a putative depression of their metabolism in ALS brain) raise the intriguing possibility of a common pathway across different genetic mutations characterized by steroids impairment, in which receptor up-regulation may correspond to an endogenous protective mechanism, and that could be targeted by available treatments like steroid hormones. In humans, mainly serum steroids have been studied in ALS patients, showing increased levels of testosterone in comparison with healthy controls, which did not decline with aging differently from controls, and that was correlated to disease progression.^22^ Other studies showed increased levels of cortisol^60^ and progesterone^61^ in ALS patients with respect to controls, but this difference appeared to be too small to be used as biomarkers. The studies, addressing CSF samples and patients with gene mutations associated with ALS, are lacking. Importantly, cases and controls at the time of the lumbar puncture (LP) did not take steroidal treatment in our study. Unfortunately, the size of the ALS group, in the current study, does not allow for properly investigating sexual dimorphism.

Interestingly, steroid hormones (corticosterone, hydrocortisone, testosterone, and estrone) were shown to affect brain metal levels (copper and zinc) and metal-binding metallothionein expression in mouse brains.^28^ In general, steroids may significantly modulate redox balance in the body,^29^ which may involve redox-active metals and selenoenzymes like GPX and TRXNRD. OPLA-DA study of the potential association of metal species and steroids showed that *N*-(8*Z*,11*Z*,14*Z*-icosatrienoyl)-ethanolamide (*m*/*z* 350.305) was associated with total Mn (Figure 2) for the ALS specimens. 3β,17β-Dihydroxyetiocholane (*m*/*z* 293.247) associated with unidentified Mn species (Mn-unknown) and relative content of Fe(III) for the controls. Additionally, for the controls, some association of these Mn and Fe species was observed for female sex hormone 2-methoxy-17β-estradiol (*m*/*z* 303.195), male steroid hormone androst-4-ene-3,17-dione (*m*/*z* 287.201), as well as other steroid species 3β,17β-dihydroxyetiocholane (*m*/*z* 293.247). Unfortunately, the literature contains only scarce results on the interplay of steroids and metal species.

Finally, we observed the difference in the loading values of different classes of lipophilic compounds, such as fatty acids, including metabolites of arachidonic acid, fatty amines, and membrane lipids, including phospholipids and acyl-carnitines. These compounds are easily ionizable in the electrospray source; thus, they are rather easily detectable using the analytical protocol used. Recently, brain lipidome was shown to play a major role in ALS pathology,^19−21^ so the role of the mentioned groups of lipophilic compounds should warrant further lipidome-targeted studies. The involvement of lipidome in ALS pathology in the current study is partially supported by the findings related to selenoenzymes GPX and TRXND, which are involved in antioxidant protection, including counteracting lipid peroxidation and ferroptosis.^46,62−64^ Another supportive argument is the potential implication of such redox-active metals as manganese and copper in ALS pathology (Table 3). In the current study, we observed a weaker association between metal species and lipids compared to metal species and steroids. However, some association was observed between Cu(II) and antioxidant selenoenzyme TRXND with membrane lipids (SM(d18:0/16:1(9*Z*)), *m*/*z* 703.575) and fatty acid acyl glycoside corchoionoside C (*m*/*z* 387.201) in the ALS cases. Interestingly, sphingomyelins, including SM(d18:0/16:1(9*Z*), were shown to be discriminatory metabolites in the Cu-chelator cuprizone-induced murine model of multiple sclerosis.^65^ Cell culture research also indicated the effect of Cu on sphingomyelins.^66^ ALS patients showed a positive correlation between copper status (blood plasma copper) and HDL–cholesterol, whereas in matched control, the association was found for both HDL- and LDL–cholesterol.^67^ Moreover, copper was found to be associated with oxidative stress of lipidic components in the rat astrocyte model of ALS.^68^ There is some evidence on the implication of iron in membrane lipid metabolism too,^69^ through lipid peroxidation and ferroptosis.^45^ Our data (Table 2) indicate the alteration in membrane lipids, lipid transport (acyl-carnitines), and arachidonic acid metabolism in ALS.

The accepted connection of disturbed redox biology with lipid peroxidation in cellular downstream particularly emphasizes the importance of further in-depth studies of the potential interplay of copper and manganese redox states with brain lipidome and steroids.

### Limitations

Our study is subject to several limitations: one significant limitation is the small sample size of the genetic ALS cohort, which is due to the rarity of ALS cases with confirmed disease-related mutations. This constraint hampers the ability to conduct subgroup analyses, such as examining the impact of specific mutations since differences in the metallome/metabolome of each mutation would be possible (e.g., zinc and copper ions are essential cofactors for SOD1 and RNA-binding proteins like FUS, whereas iron promotes the aggregation of C9ORF72-derived dipeptide repeat proteins, together with copper) as well as distinguish between disease-causing and disease-modifying mutations (i.e., *ATXN2* vs other mutations). Then, we could not evaluate the influence of individual factors like sex or age on the observed results. In addition, the body weight index was not available for analysis. Further, only very small sample volumes per sample were available. Therefore, after the metallomics and nontargeted metabolomics analysis of the CSF samples, the quantities of the remaining samples (both ALS and control groups) were too low and running short to confirm the identity of the compounds using targeted MS/MS analysis, which is a principal limitation of the current study.

### Strengths

The unmistakable strength of this study lies in the combination of the data from selenium and redox speciation techniques combined with nontargeted FT-ICR-MS-based metabolomics, each performed in a sample matrix from inside the brain compartment, namely, CSF, taken from both, ALS patients with ALS-related mutations (which is extremely rare, since most ALS cases do not exhibit identified genetic mutations), confirmed by genetic screening, and from matched control individuals. Finally, the entire data were evaluated using advanced statistical methods.

## Conclusions

In this study, we successfully showcased the effectiveness of integrating metabolomics and metallomics in investigating potential pathological alterations within the CSF of individuals with ALS compared to neurologically healthy controls. Through multivariate analysis, we were able to set the strongest relations between the two different sets of data experiments, confirming already some knowledge discussed previously in the literature and opening new scenarios for possible new hypotheses.

Reduced steroids, including sex hormones, as well as copper and manganese species were found to be the most prominent features of ALS CSF samples. This indicates a potential impairment of sex hormone pathways in the ALS-affected brain, as reflected in the CSF. The findings of the current study need to be further elaborated in larger cohorts with targeted studies, this corresponds, first of all, to the involvement of CSF lipidome in the ALS pathology.

## Materials and Methods

### Study Population

We analyzed metabolites in CSF samples of 7 ALS cases with disease-related mutations, assessed through gene screening (Table 1), and 13 controls. All patients were tested negatively against HIV, hepatitis B, and C according to German Biosafety regulations before enrollment. It must be noted that such patients belong to a highly specific and extremely rare patient population, which explains the low case number available for this study. Previously, the same set of samples was used to assess the exposure to Se and redox-active metals (Cu, Fe, and Mn).^4,30^ In brief, the CSF specimens were provided by three major Italian ALS Referral Centers (Milan, Modena, and Rome) from Italian patients. The patients were diagnosed with definite or probable ALS since 2002 and were identified to carry an ALS-related gene mutation. All patients underwent LP at diagnosis.^70^ After performing all necessary clinical chemistry, trace element analysis, and chemical speciation, only the samples, which are not contaminated with blood and still have at least 0.5 mL of CSF available, were enrolled for this study. The demographic characteristics of ALS patients are summarized in Table 1. The age- and sex-matching for the controls is demonstrated in Table S1 (Supporting Information). The study was approved by the Azienda Ospedaliero Universitaria of Modena Ethics Committee (protocol number 85/15, dated 07 July 2015).

### Gene Sequencing

Search for *C9ORF72* gene expansion was performed by repeat primed polymerase chain reaction (PCR) Amplidex PCR/CE C9ORF72 Kit (Asuragen Inc., Austin, TX, USA). PCR and genetic sequencing were used to detect *SOD1*, *VCP*, *TARDBP*, and *FUS* gene mutations (Big-Dye Terminator v3.1 sequencing kit, Applied Biosystems Inc.; ABI Prism 3130 genetic analyzer).^71,72^*TUBA4A* and *ATXN2* gene analyses were performed as previously reported.^73,74^ Control samples came from patients who underwent LP due to a suspected neurological condition that was later unconfirmed after complete clinical and instrumental investigations based on the criteria proposed by Michalke et al.^38^

### Samples Collection and Storage

CSF samples were obtained through LP, according to an established procedure at the three aforementioned Neurology Centers, as previously reported.^4^ In brief, after localizing L3–L4 interspace, skin swabs, an antiseptic solution, and an adhesive sterile drape were used to create a sterile field on the patient. CSF was withdrawn through a 20 gauge needle after a local anesthetic, and 6–8 mL of CSF were initially collected in sterile polypropylene tubes. The samples were immediately frozen and stored at −80 °C. To maintain blindness, all collected specimens were subjected to a rigorous labeling system. Any clinical or demographical data were removed. Anonymized samples were transported by air courier on dry ice to the Helmholtz Zentrum München and kept continuously frozen at −80 °C until use.

## Chemicals

The chemicals used in this study were as follows: methanol (hypergrade for LC–MS, LiChrosolv, Merck Chemicals), l-arginine from Sigma-Aldrich (>98% purity, St. Louis, MO, USA), and formic acid (for MS, Honeywell Fluka). Chemicals used for metallomics study were already described in previous reports.^30,75^

### Metabolic Profiling

#### Sample Preparation

Prior to direct input (DI) FT-ICR-MS analyses, a solid-phase microextraction (SPME) was used based on a protocol previously reported.^33,76^ In brief, frozen CSF samples were thawed on ice and vortex-mixed before treatment. C4 ZipTip SPME cartridges from Agilent Technologies (Santa Clara, CA, USA) were used for matrix separation and desalination of the CSF samples. 50 μL of CSF was taken and diluted with 50 μL of purified water (MilliQ). The cartridges were preliminarily primed with methanol and 2% formic acid in water. The diluted sample (100 μL) was loaded onto the cartridges (on ice), rinsed with 2% formic acid solution, and eluted with 100 μL of methanol (on dry ice). All extracts were diluted in methanol 1:5 (v/v %) in methanol before analysis (final dilution factor 1/10).

#### FT-ICR-MS Measurement

The measurement of metabolic profiles was performed using an ultrahigh-resolution direct infusion FT-ICR-MS spectrometer SolariX (Bruker Daltonics, Bremen, Germany), equipped with a 12-T superconducting magnet (Magnex Scientific, Yarnton, United Kingdom) and an electrospray ionization (ESI) source Apollo II (Bruker Daltonics, Bremen, Germany), as previously described by Willkommen et al.^33^ An l-arginine solution (3 mg/L in methanol) was used for the external calibration of the mass spectra with errors below 0.1 ppm. All measurements were undertaken in positive ionization mode and ion accumulation time of 300 ms was applied in the argon-filled collision cell for enhanced sensitivity. The resolution was on average *R* = 400,000 at *m*/*z* 400 with a recorded time domain transient size of 4 MW and length >1.6 s for each FT-ICR-MS acquisition, enabling an excellent signal differentiation on a molecular level. ESI source parameters were set as follows: an injection flow rate of 2 μL/min, a source operating temperature of 180 °C, a nebulizer gas flow rate of 2 L/min, and a dry gas flow rate of 4 L/min. The spectra were recorded in an *m*/*z* range of 147–1400 Da. For the generation of each mass spectrum, 300 scans were acquired.

### Metallomics Profiling

Quantification of total Se, Fe, Mn, and Cu in CSF and speciation of these elements were performed as reported in previous publications.^4,30,75^ In brief, total concentrations of Cu, Fe, Mn, and Se in CSF were quantified using a dynamic reaction cell mass spectrometer NexIon DRC from PerkinElmer (Rodgau-Jügesheim, Germany). ^103^Rh (1 μg L^–1^) was used as an internal standard for all elements. Ion exchange chromatography using a Beckman System Gold 127NM Solvent Module (Beckman Coulter Biomedical, Munich, Germany) high-performance liquid chromatography system, equipped with 9725i PEEK injection valve from Rheodyne (Sigma-Aldrich, CT, USA) and degasser Degassex Model D6-4400 (Phenomenex, Darmstadt, Germany) was used for the trace element species separation.^30,75^ The following columns were used: a Dionex AS-11 ion exchange column (250 × 4 mm i.d.) for Se species and an analytical cationic column Dionex IonPac CS5A RFIC 4*250 mm with a guard column Ion-PacCG5A (all from Thermo Scientific, Idstein, Germany) for redox species of Fe, Cu, and Mn. Double focusing sector field ICP–MS (spectrometer Element 2, Thermo Scientific, Bremen, Germany) was used for the detection of Fe, Cu, and Mn species at medium resolution mode (*m*/Δ*m* = 4000) and Se in high-resolution mode (*m*/Δ*m* = 10,000).^4,30,75^

### Data Analysis

Initially, our metabolomics dataset consisted of 7695 MS features. To refine and focus our analysis on metabolites of interest, we applied a filtering step to exclude features associated with the presence of multiple heavy atoms, such as sulfur or phosphorus. This resulted in a reduction of the dataset, narrowing it down to 1745 features. For the metallomics measurements, we used the total concentrations and the species characterization. Here, all the variables are reported that were included in the statistical evaluation (Table S3, Supporting Information). To enhance the comprehensiveness of our analysis, we integrated the data from metallomics and metabolomics, allowing us to obtain two distinct types of measurements for each sample. By combining information from both fields, we gained a more comprehensive understanding of the molecular landscape associated with ALS.^32,77^ The dataset was unit variance-scaled and analyzed through an OPLS-DA. The *Y* variables were set to dichotomous values: 0 = control, 1 = ALS. The model was tested for the ability to classify and discriminate. Hotelling’s T2 test (95%) was applied to prohibit the influence of strong outliers on the models. The classification performance was evaluated using sevenfold cross-validation. As a result, the model inferred strongly interrelated masses and metallomics variables, and a list of the most important masses were defined, choosing the highest loadings values. We isolated them in a list of masses (Table S4) that we denominate core metabolome.^33^ For each variable, we have calculated the *p*-values and the false discovery rate. Moreover, we added the AUC values with the fold change values. Those masses drive the main separation between the two classes. By further studying and evaluating such lists of correlated variables, we found them to show a particular alteration in ALS patients from a biological point of view. Moreover, we focused on the reduced list of biomarkers to find a possible biological interpretation and insight into the metallomics and metabolomics relations. Another statistical model was applied, sparse PLS-DA. This confirmed the relations between the two datasets and the ability to separate the case from the control groups. Those elaborations were done in SIMCA 13.0.3.0 (Umetrics, Umeå, Sweden) and with reportROC and MixOmics packages (RStudio Version 1.0.136–2009–2016, RStudio, Inc., Boston, MA, USA).

For the metabolomics data library, searches were executed using an R script based on the MassTRIX approach.^78^ KEGG (https://www.kegg.jp/kegg/kegg2.html) and HMDB (https://hmdb.ca/) were used for the identification of the metabolites and metabolite categorization.

## Supporting Information Available

Case–control age- and sex-matching; intensities of Fourier transform mass spectrometric components of CSF from ALS cases and controls (control); metallomics species considered in this study; and core metabolites for the CSF samples of ALS patients with disease-related mutations (XLSX)
